# Structural evolution of metatitanic acid and iron removal during hydrolysis of industrial TiOSO_4_ solution

**DOI:** 10.1038/s41598-023-35741-0

**Published:** 2023-05-25

**Authors:** Congxue Tian, Guangqiang Ma, Hongwei Ge

**Affiliations:** Panzhihua University, Panzhihua, 617000 Sichuan People’s Republic of China

**Keywords:** Chemistry, Materials science

## Abstract

The ferrous ion content of metatitanic acid affected the whiteness, purity and applications of TiO_2_, controlled by the hydrolysis conditions and metatitanic acid structure. The structural evolution of metatitanic acid and ferrous ion removal was investigated by hydrolyzing the industrial TiOSO_4_ solution. The hydrolysis degree was conformed to Boltzmann model with good fitting. TiO_2_ content of metatitanic acid gradually increased as hydrolysis proceeding due to its stronger compact structure and weaker colloidal property, caused by the aggregation and adjustment of the precipitated particles. At lower TiOSO_4_ concentration, the crystal size increased significantly, lattice strain decreased, and average particle size constantly adjusted and reduced. The micropores and mesopores were mainly formed by aggregating and stacking of primary agglomerate particles, bonded and filled with sulfate and hydroxyl. The ferrous ion content decreased linearly with the increase of TiO_2_ content, and reducing moisture content of metatitanic acid was an effective way to reduce Fe content. This would save more water and energy consumption, help to improve the clean production level of TiO_2_.

## Introduction

Titanium dioxide (TiO_2_) is the third largest inorganic chemical, due to its excellent physical and chemical properties, it is widely used in many fields, such as coatings, plastics, rubber, etc.^1–3^. The industrial production of TiO_2_ mainly includes the sulfate process and the chloride process. The titanium resource reserves in Panzhihua Xichang region of China are the largest in the world. It is more suitable to produce TiO_2_ by the sulfate process due to its higher calcium and magnesium content. Hydrolysis of industrial TiOSO_4_ solution is the core step in the preparation of titanium dioxide by sulfate process. It will go through many complex physiochemical processes, including polymerization reactions of hydroxyl bridge and oxygen bridge connection structure to form the nuclei, TiO^2+^ ionic reaction to promote crystal growth and aggregation, colloidal particle formation, condensation and precipitation, and finally the formation of metatitanic acid (*MA*) particles^4^. When TiOSO_4_ solution is hydrolyzed, a large number of colloidal microcrystals (7–8 nm) were first formed from crystalline TiO^2+^ ions, then these microcrystals aggregated and formed the primary aggregate agglomerates (60–100 nm). The size and distribution of these primary agglomerates were important key factors determining TiO_2_ structure and pigment properties. The primary agglomerates re-aggregated to form the secondary aggregates with a particle size of 1–2 μm, which was called *MA* precipitation^5,6^. As there was a large amount of ferrous sulfate in the industrial TiOSO_4_ solution, the mass ratio of Fe-to-TiO_2_ (Fe/TiO_2_) had strict requirements in order to meet the TiO_2_ quality requirements. The ferrous sulfate played an important role in increasing the relative density, viscosity and total ion concentration of the solution, and it would also affect the hydrolysis process and the precipitation of *MA*. The impurities such as ferrous ion (Fe^2+^) and SO_4_^2-^ affected the hydrolytic conversion, structure and composition of *MA*, they would substantially affect the impurities adsorbed by the hydrated TiO_2_, the adsorption capacity increased with increase of impurity concentration, and at the same time, the agglomeration of the primary particles would increase^7^. Ferrous ion concentration influenced the conversion degree of TiOSO_4_ to hydrated TiO_2_, but did not influence the mean size of the hydrated TiO_2_ crystallites^8,9^. Researchers had widely investigated the influences of hydrolysis conditions of the TiOSO_4_ solution hydrolysis system on hydrolysates and TiO_2_^10–14^. Titanium dioxide white pigment with narrow particle size distribution and good performances could also be prepared from the un-enriched industrial TiOSO_4_ solution by adjusting the hydrolysis conditions^15,16^. Whiteness was a very important index of TiO_2_ pigment, and ferrous ion content was the most important influencing factor. The less the ferrous ion content was, the higher the whiteness of TiO_2_ was. The aims of adjusting hydrolysis conditions, filtration, rinsing and other processes were to control the ferrous ion content in *MA*, so as to obtain good quality of TiO_2_. The increase of ferrous ion concentration would affect the formation of *MA*, reduce the reacting activity of TiO^2+^ ion, and influence the crystallization and particle size of *MA*. The composition and structure of *MA* would change with hydrolysis process, which would also affect the adsorption of water, sulfate and impurities, affect the crystal transformation and crystal growth in the subsequent calcination process, ultimately affected the purity of *MA* and product performances of TiO_2_. The problems were that the structural changes of *MA* during the hydrolysis process and its impact on the content of ferrous ions made it difficult to accurately control the quality and impurities content of *MA*. However, there were few reports about this.

The aim of this work was to investigate the effects of the structural evolution of *MA* during hydrolysis of industrial TiOSO_4_ solution on the ferrous ion content in *MA*, which would be of great significance to control the hydrolysis conditions to obtain hydrolyzed *MA* with good composition and structure, so as to ensure the quality and performances of titanium dioxide pigment.

## Experimental

### Hydrolysis

Thermal hydrolysis for the industrial TiOSO_4_ solution as raw material was carried out by extra-adding seeded hydrolysis method. The TiOSO_4_ solution was an industrial grade raw material, and the water used was deionized water. The typical composition of industrial TiOSO_4_ solution was with the total TiO_2_ concentration of 194 g/L, *F* value of 2.03 (*F* value meant the mass ratio of free sulfuric acid and sulfuric acid combined with Ti^4+^ (TiOSO_4_) to TiO_2_, as with free sulfuric acid concentration of 1.59 mol/L), Fe/TiO_2_ ratio of 0.30 (as with ferrous ion content of 1.04 mol/L), Ti^3+^ concentration of 1.5 g/L. The hydrolysis reacted in a four port round bottom flask with heating, stirring and condensation reflux, the typical hydrolysis was conducted as the following procedure. The extra-adding seed, with amount of 2.1% and concentration of 144 g/L, was added to the above industrial TiOSO_4_ solution which was pre-heated to 96 °C, and the starting point of hydrolysis time was the completion of feeding. After adding, the hydrolysis slurry was uniformly mixed and heated to the first boiling point at 107 °C, then kept in a slightly boiling state. When the hydrolysis slurry turned into gray color at 60 min after feeding, the hydrolysis reaction entered the ageing stage by stopping heating and stirring for another 30 min. After ageing, the slurry was heated again to the second boiling point at 108 °C in 17 min, then also keeping in slightly boiling state. The dilution water was slowly added to the hydrolysis system with volume ratio of 2% in 15 min at hydrolysis time of 227 min. The hydrolysis reaction was stopped at 287 min. At different hydrolysis time, 200 mL hydrolysis slurry was taken out from the hydrolysis system and filtered without washing, the obtained sample called wet MA. Metatitanic acid powders were obtained by grinding after drying the MA filter cake at 100 °C for 6 h, and the obtained *MA* powders were called dried *MA*.

### Characterization

The hydrolysis degree was determined by measuring the residual TiO_2_ in the filtered TiOSO_4_ solution, TiO_2_ content by determining the TiO_2_ content of the dried *MA*, according to the standard ISO 591-1:2000 ‘Titanium dioxide pigments for paints’, by using the ammonium ferric sulfate oxidation–reduction titration method. A X-ray diffractometer (*X’ Pert*^3^* Powder*, PANalytical) was used to determine the crystal structure for the wet *MA* samples, by using a tube voltage of 40 kV, a tube current of 40 mA, scanning angle from 20° to 70°, with a scanning step size of 0.0133 and 0.1 s/step. And the anatase grain size *L*_*(101)*_ for crystal plane *(101)* was calculated according to the *Scherrer* equation^17^. The lattice strain of *MA* in the *C*-axis direction was obtained by refining the structure through the reflex module of MS software. The SEM morphologies of the *MA* samples were obtained by the field emission scanning electron microscopy (*Sigma 300*, Zeiss, Germany), by using a test voltage of 30.0 kV, WD of 8.0 mm and secondary electronic signal. Particle size distribution (*PSD*) was determined by a Malvern particle size analyzer (*Mastersizer 2000*, Malvern), by using a wet dispersion system, helium neon gas laser light source (633 nm) and blue assisted light source (466 nm), with a shading ratio of 13*%*. BET surface area (*S*_*BET*_) and the pore size distribution was measured on the surface and pore size distribution analyzer instrument (*Autosorb-iQ3*, Quantachrome, USA), the sample was first degassed under vacuum conditions at 200 °C for 6 h, and then measured using nitrogen as the adsorbate. The Raman spectra for *MA* samples were obtained from a micro confocal Raman spectroscopy by using laser with wavelength of 532 nm for measurement (*inVia*, Renishaw, UK), by using a 532 nm laser for testing, static scanning, wavenumber range of 87.93–959.47 cm^−1^, exposure time of 10 s, exposure intensity of 5%. The FT-IR test and analysis were carried out on the infrared spectrometer (*Nicolet-380*, Thermo, USA), by using *KBr* compression method, with a wavenumber range of 400–4000 cm^−1^, a maximum resolution of 0.05 cm^−1^, and the scanning speed of 20 cm^−1^/min. The ICP-OES (*iCAP 6300*, Thermo Fisher, USA) was used to determine the Fe content of the *MA* samples, by using the standard curve method, determined the characteristic emission intensity of ferrous ions at 259.940 nm for the tested sample, and then determined its Fe content through the standard curve. The binding energies for *MA* samples were determined by an X-ray photoelectron spectroscopy (*XPS*) (*XSAM-800*, Kratos), by using Al *Kα* (1486.6 eV) X-ray gun operation, 12 kV × 15 mA, the analytical instrument adopted high magnification, fixed reduction ratio, high-resolution mode, and the analyzer was calibrated with Au and Ag standard samples.

## Results and discussion

**Figure 1:**
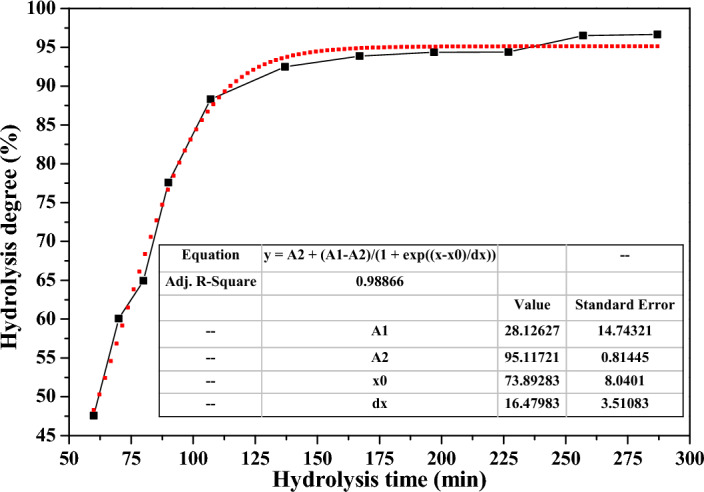
The variation curve for the hydrolysis degree.

The *MA* samples were taken from the hydrolysis slurry for filtration and separation to determine its structure and composition as the hydrolysis proceeding. Since the initial formed *MA* particles were too small to be separated, the *MA* samples were taken from the gray point (that is, the gray point was that the hydrolytic slurry turned into steel gray color at 60 min) and after. The hydrolysis degree of industrial TiOSO_4_ solution, TiO_2_ content, crystal size (*L*_*(101),MA*_), lattice strain, average particle size (*D*), BET surface area (*S*_*BET*_), pore diameter, ferrous ion content (Fe %) for the *MA* samples were listed in Table 1. The variation curve of hydrolysis degree at different hydrolysis time was showed in Fig. 1.

**Table 1:** Hydrolysis degree, compositions and structures for metatitanic acid at different hydrolysis time.

No.	Hydrolysis time (min)	Hydrolysis degree (%)	Wt,_TiO2_ (%)	L_(101) ,MA_ (nm)	Lattice Strain (%)	D (µm)	Diameter distance ratio*	S_BET_ (m^2^/g)	Pore diameter (nm)	Fe (%)
1	60	47.57	51.99	12.7	0.541	1.54	0.99	268	3.4	2.61
2	70	60.07	63.06	13.1	0.539	1.58	1.01	274	3.4	1.82
3	80	64.94	67.61	13.4	0.537	1.65	1.01	277	3.4	1.27
4	90	77.59	73.00	14.4	0.570	1.57	1.01	265	3.4	1.00
5	107	88.30	73.06	15.0	0.563	1.58	1.03	286	3.4	0.87
6	137	92.48	75.39	15.6	0.559	1.62	1.00	311	3.4	0.46
7	167	93.87	79.21	15.7	0.551	1.67	1.04	309	3.4	0.11
8	197	94.35	79.99	21.4	0.545	1.75	1.05	298	3.4	0.10
9	227	94.40	80.09	22.4	0.552	1.48	1.03	307	3.1	0.09
10	257	96.50	78.31	22.8	0.536	1.36	1.04	295	3.4	0.28
11	287	96.65	79.15	23.8	0.524	1.31	1.05	285	3.4	0.20

*The *Diameter distance ratio* was the degree of dispersion of particle size distribution, represented by the calculated result of (D_90_–D_10_)/D_50_ as for the measured particle size.

The variation curve of hydrolysis degree could be divided into two stages, as shown in Fig. 1. The first stage from the gray point to 125 min was the rapid hydrolysis stage by first derivative of the curve, characterized by its large change in the hydrolysis degree and large precipitation of *MA*. The second stage from 125 min to the end of hydrolysis was the maturation stage, the hydrolysis degree changed slowly and tended to be stable and balanced gradually, and reached at the highest value of 96.65% at the end of hydrolysis process. The dilution water was added at 227 min in order to improve the hydrolysis degree, which could make the overall hydrolysis degree increase by 2%, so as to meet the hydrolysis degree requirements. The change of hydrolysis degree conformed to Boltzmann model, and the correlation coefficient *R*^2^ was of 0.98866, indicating that the results had a good fitting degree. The fastest hydrolysis rate was at 74.0 min when the hydrolysis curve was differential treated, and then the hydrolysis rate gradually slowed down. In the first hydrolysis stage, it was mainly controlled by the surface reaction of *MA* particle and grain growth as there were a lot of TiO^2+^ ions in the high concentration of TiOSO_4_ solution to form the *MA* crystals. While in the second hydrolysis stage, the reaction was mainly controlled by diffusion process due to the low concentration of TiO^2+^ ions to increase the crystal growth, and the reaction rate was relatively slow.

As the hydrolysis time increased, the TiO_2_ content of the dried *MA* samples increased rapidly at first and then slowly after the hydrolysis time of 107 min, as shown in Table 1. After that it was stable at about 73–80% in the subsequent process. This indicated that the structure of *MA* became more and more compact with the extension of hydrolysis time, and the adsorbed water content in *MA* decreased, showing that the colloidal properties of *MA* was weakened. At the later hydrolysis stage, some fine *MA* particles were precipitated due to a small amount of TiOSO_4_ solution remaining in the hydrolysis slurry and the dilution water adding. These fine *MA* particles would absorb more water and impurities, and aggregate with the previously precipitated *MA* particles because they were ultra-fine and had high surface activity, resulting in TiO_2_ content decreasing. In addition, the structure and TiO_2_ content would be also affected by the re-aggregation and particle adjustment of the precipitated *MA* particles during the hydrolysis process.

**Figure 2:**
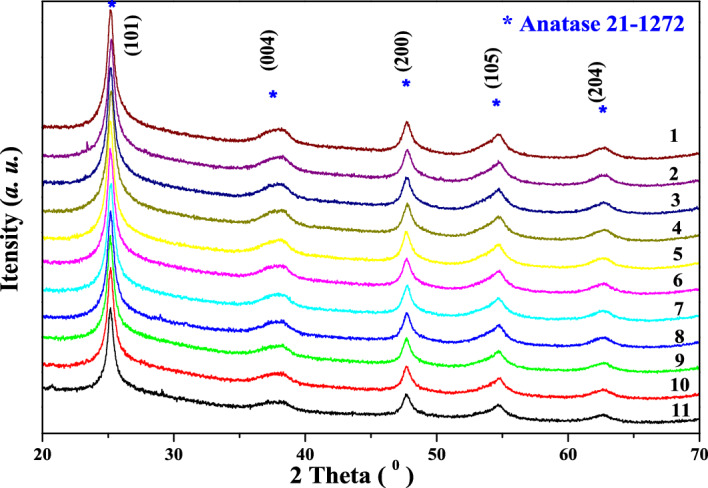
XRD patterns for the metatitanic acid samples.

The XRD patterns for the wet *MA* samples were showed in Fig. 2, clearly agreeing with the main diffraction peaks of the standard anatase phase (*JCPDS* 21-1272), and anatase structure of would be obtained after calcination process. This was because the metatitanic acid formed in the SO_4_^2-^ system was prone to obtaining anatase TiO_2_ like crystal structure. As the hydrolysis time increased, the diffraction peak intensity gradually increased and the diffraction peak became narrow and sharp, indicating that the *MA* crystallinity gradually increased. The calculated crystal size of anatase face *(101)* for the *MA* samples ranged from 12.7 to 23.8 nm, also showed the *L*_*(101)*_ increased with the increasing of the hydrolysis time, consistent with the aforementioned *XRD* analysis. The crystal size increased significantly from 167 to 197 min, indicating that the crystal size evidently grew when the concentration of TiOSO_4_ solution was low, consistent with the above analysis of the slow hydrolysis stage. The high angle diffraction peak gradually shifted to the low angle as the hydrolysis proceeding in Fig. 2, indicating that the unit cell parameters gradually became larger and the crystal plane spacing increased, mainly a reflection of lattice distortion caused by macro residual stress, which might be caused by the tensile stress during the crystal growth of *MA*. This also showed that the initial formed *MA* had loose structure and high stress. With the crystal growth and hydrolysis process, the crystal structure of *MA* was constantly adjusted, and the corresponding stress gradually decreased. The lattice strain for the *MA* samples showed a slightly decreasing trend, mainly fluctuating at about 0.54%. At the late hydrolysis stage, the lattice strain increased due to the newly formed ultra-fine *MA* particles. The minimum lattice strain of *MA* was at the end of hydrolysis, with the value of 0.524%, which indicated that the *MA* crystal was constantly adjusted to gradually reduce stress and maintain low energy during the hydrolysis process.

The *D* for the wet *MA* samples was also listed in Table 1, ranged from 1.31 to 1.75 μm, increased firstly and then decreased as the hydrolysis proceeding. The diameter distance ratios of the *MA* particles were about 1.00, and the overall changes were not significant, indicating that the width of particle size distribution was relatively close during different hydrolysis time. Metatitanic acid particles were easy to aggregate due to their smaller crystals and higher surface energy, and the measured* D* mainly corresponded to the secondary aggregates, which was formed by the primary agglomerates composed of many *MA* crystal particles. With the crystal size of *MA* increasing, the primary agglomerates would be smaller, and the secondary aggregates would be larger, which was consistent with the changing trend of *D*. Due to the addition of dilution water, some fine *MA* particles were formed, and their structure and aggregation state were changed, resulting in the reduction of the *D* for the *MA* samples.

**Figure 3:**
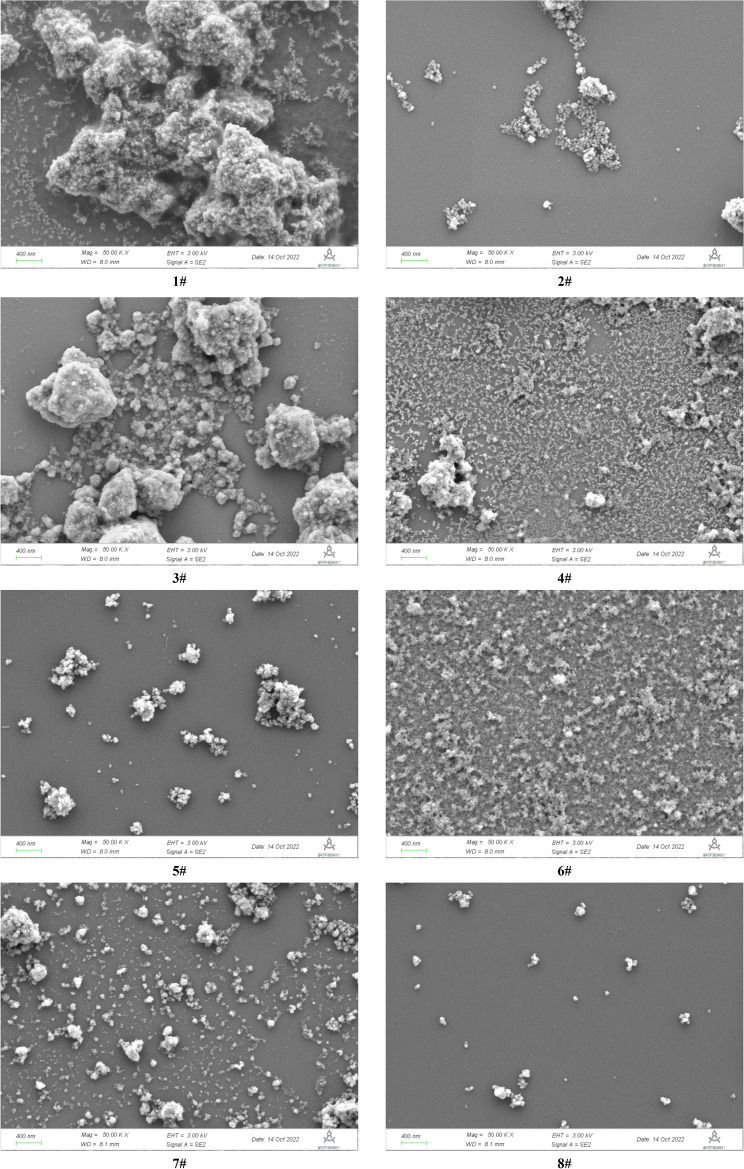

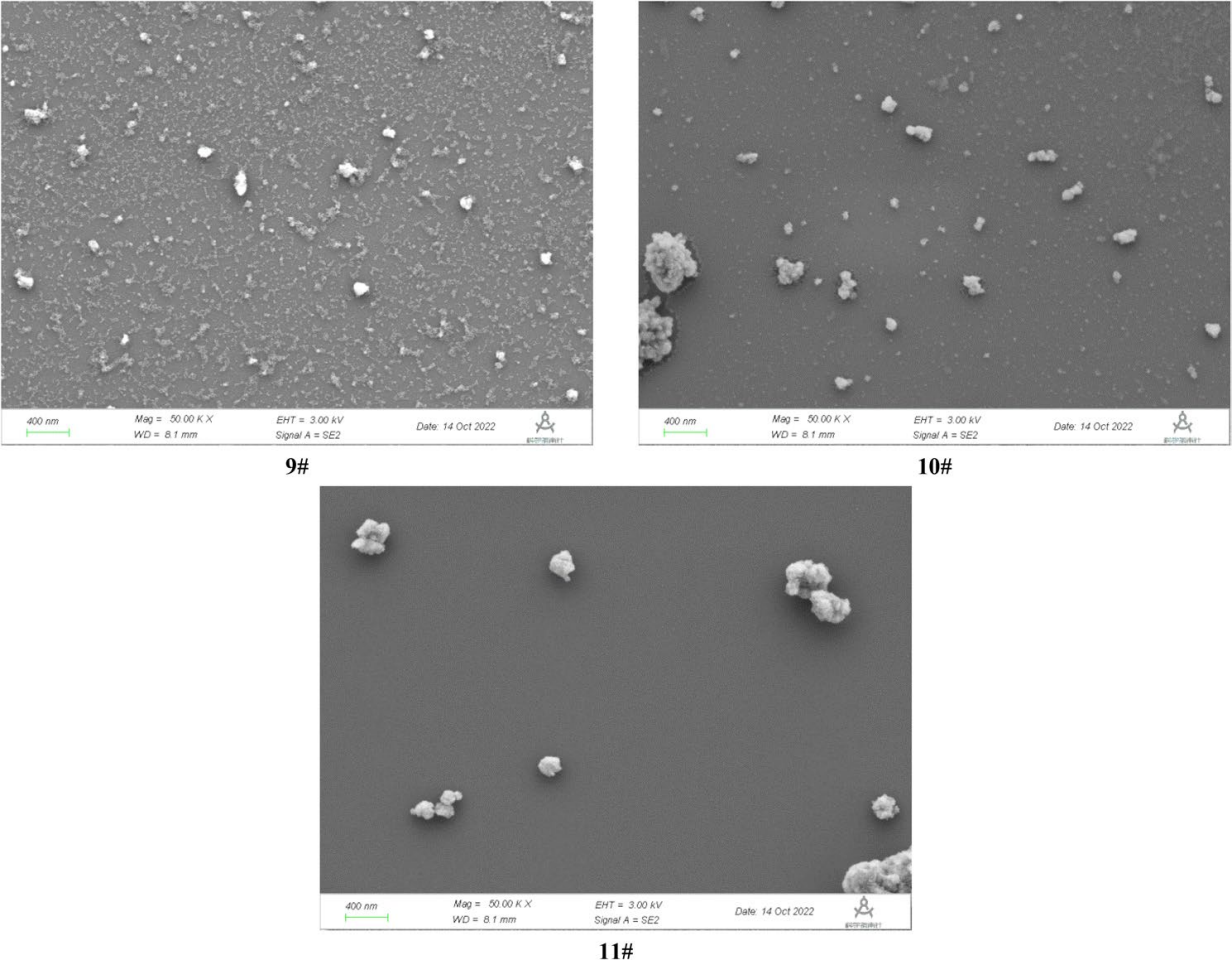
SEM photographs for the metatitanic acid samples (50kX).

The *SEM* photographs for the dried *MA* samples were showed in Fig. 3. After ultrasonic dispersion in ethanol solvent, the *MA* samples mainly existed in the form of aggregates, while containing some small particles, and the aggregates were formed by finer particles. The size of dispersed fine particles was at about 50–70 nm, which corresponded to the primary agglomerates of the *MA*. The photographs also showed the different sizes and aggregation states of the precipitated *MA* particles at different hydrolysis time. The secondary nucleation promoted the formation of crystal clusters, then formed the primary agglomerates through surface nucleation, and formed the micron aggregates by physical forces^5^.

**Figure 4:**
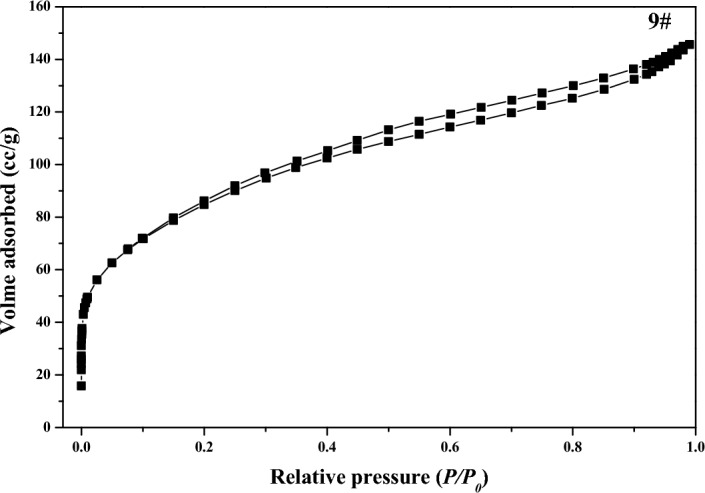
Nitrogen isotherms for sample 9.

**Figure 5:**
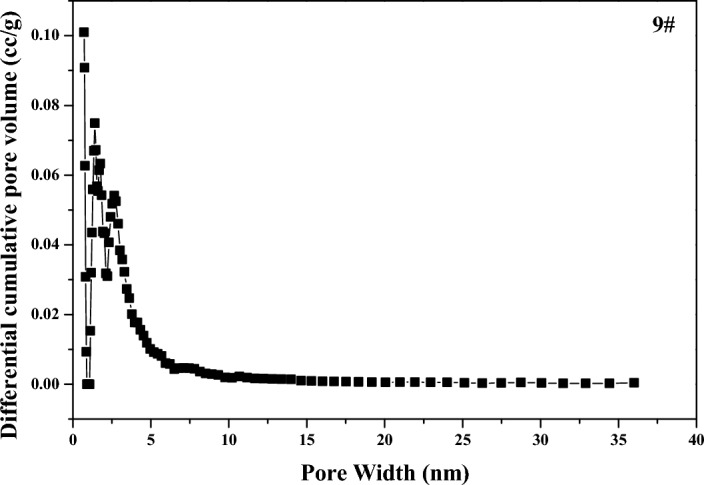
Pore size distribution curve for sample 9.

The *S*_*BET*_ of the dried *MA* samples ranged from 268 to 311 m^2^/g in Table 1. With the hydrolysis time increasing, the *S*_*BET*_ first gradually increased from 268 m^2^/g at the gray point to 311 m^2^/g at 137 min, then gradually decreased to 285 m^2^/g at the end of hydrolysis. As the hydrolysis proceeding, the pore diameter of *MA* samples mainly fluctuated at about 3.4 nm, and the overall change was very small. The *N*_2_ adsorption–desorption isotherms for sample 9 was close to the type IV in Fig. 4, there was a hysteresis loop because the adsorption desorption process was not completely reversible, indicating that there were pore structures in the metatitanic acid aggregates. As the relative pressure range of hysteresis loop was wide, which indicated that its pore size distribution range was wide, distributing in micropores and mesopores. The pore size distribution curve by *DFT* method for sample 9 was shown in Fig. 5, the average pore diameter was 3.1 nm, and the most probable pore size in the microporous range was 1.4 nm, the most probable pore size in the mesoporous range was of 2.7 nm, consistent with the previous analysis of the hysteresis loop.

**Figure 6:**
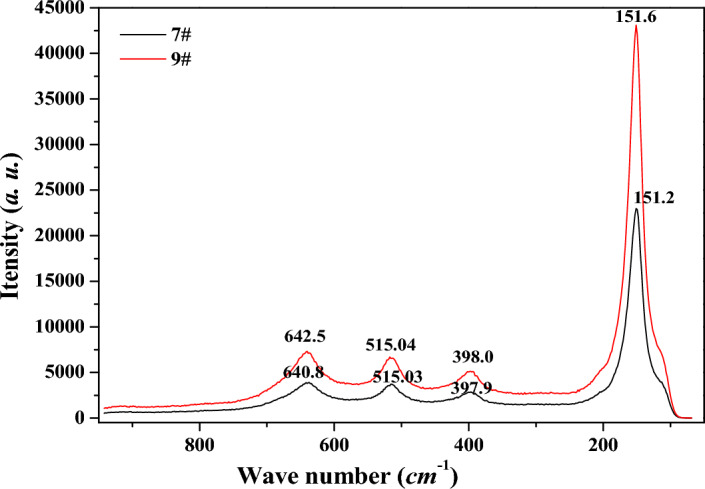
Raman spectrum for sample 7 and sample 9.

**Figure 7:**
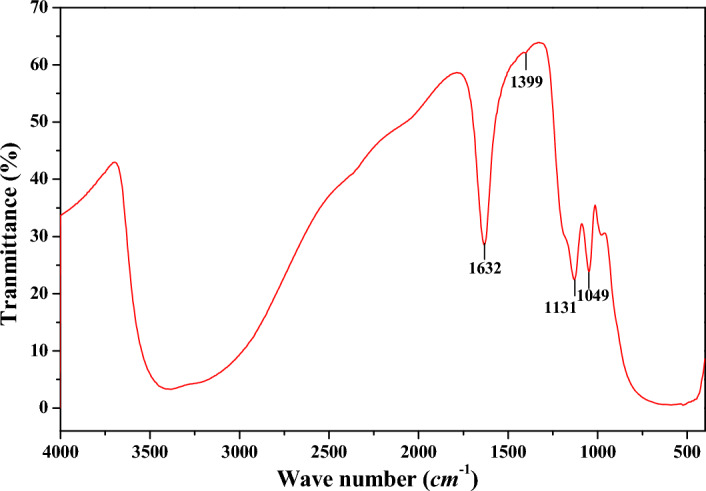
FT-IR spectrum for sample 9.

The Raman spectra for sample 7 and sample 9 were showed in Fig. 6, and spectral deconvolution was used to determine the peak position and width, and the peak positions were consistent with the Raman shift of anatase TiO_2_. And the phonon confinement model was used to explain the broadening and shifts of the Raman line shapes^18,19^. As hydrolysis proceeding, the peak positions shifted to the higher wave number position, and its corresponding peak width also narrowed, indicating that the crystal size of the *MA* sample increased, consistent with the previous XRD analysis. The infrared absorption of *MA* samples at different hydrolysis time was similar, and the *FT-IR* spectrum for sample 9 was showed in Fig. 7. The absorption peak at wave number 1632 cm^−1^ corresponded to the bending vibration of the physically adsorbed molecular water H–O–H, and the absorption shoulder peak with a wide range of 3400–3600 cm^−1^ corresponded to the stretching vibration of the sample surface hydroxyl O–H^20^. The peaks at 1049 cm^−1^ and 1131 cm^−1^ were characteristic absorption peaks of bidentate coordination sulfate ion^21,22^, which would combine with Ti–O bond to form the corresponding pore structure. The micropores and mesopores formed by *MA* were mainly formed by the aggregation of primary agglomerate particles in different sizes, bonding and filling with sulfate and water.

**Figure 8:**
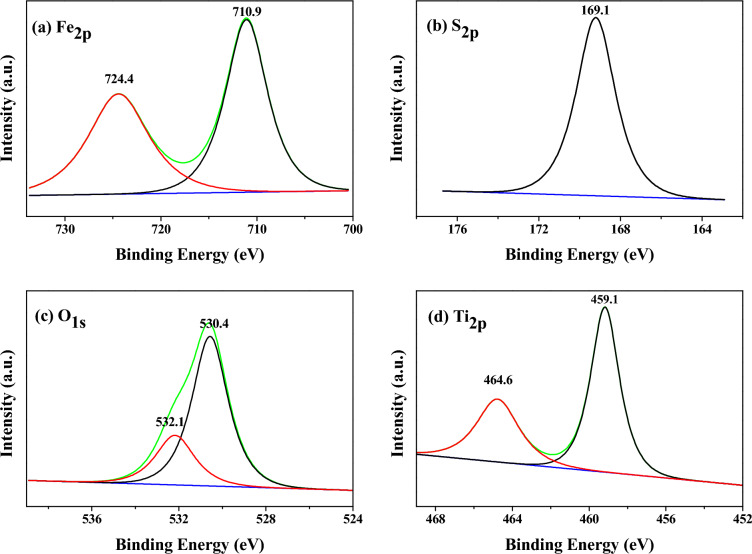
XPS fitting spectrum for sample 9 (**a**) Fe_2p_; (**b**) S_2p_; (**c**) O_1s_; (**d**) Ti_2p_.

The XPS fitting spectrum for sample 9 was showed in Fig. 8. The ferrous ions were oxidized to ferric ions in *MA* samples after long-term exposure to air. The *Fe*_*2p*_ fitting spectrum consisted of two peaks in Fig. 8a, corresponding to Fe^3+^_2p3/2_ and Fe^3+^_2p1/2_ energy levels, the binding energies were 710.9 eV and 724.4 eV respectively. Compared with standard pure Fe_2_O_3_ (the binding energies of Fe^3+^_2p3/2_ and Fe^3+^_2p1/2_ energy levels were 710.7 eV and 724.3 eV), the positive shifts of 0.2 eV and 0.1 eV might be the result that Fe^3+^ entered the crystal lattice of H_2_TiO_3_, formed the Ti–O–Fe bond and changed the chemical potential and polarity^23^. The Fe (II) ions in the near surface region of the system had lower potential energy for both dry and hydrated surfaces^24^, which was easy to enter the metatitanic acid surface for bonding, thus forming the corresponding bond valence structure. During the hydrolysis process, ferrous ions first diffused to the interface layer of *MA* precipitation through the main TiOSO_4_ solution, then diffused to the surface of *MA* through the interface layer, and formed the corresponding Ti–O–Fe structure after adsorption and bonding. And these ferrous ions in the bonding state were difficult to remove. There was only a single peak in the *S*_*2p*_ energy level fitting map in Fig. 8b, and its binding energy was 169.1 eV, indicating that sulfur only existed in the form of S^6+^. The two peaks of the O_1s_ energy levels in Fig. 8c were located at 530.4 eV and 532.1 eV, corresponding to the lattice oxygen, surface hydroxyl, and the O_1s_ energy level in the chemisorbed water^25^. The two peaks of the Ti_2p_ in Fig. 8d were located at 459.1 eV and 464.6 eV, corresponding to the Ti^4+^_2p3/2_ and Ti^4+^_2p1/2_ energy levels. Compared with the pure anatase TiO_2_ (458.1 eV and 463.8 eV), there was a positive shift, indicating that the Ti^4+^ ion for sample 9 had greater positive electricity due to the strong induction effect of the SO_4_^2−^/TiO_2_ chelating bidentate coordination structure^26^.

**Figure 9:**
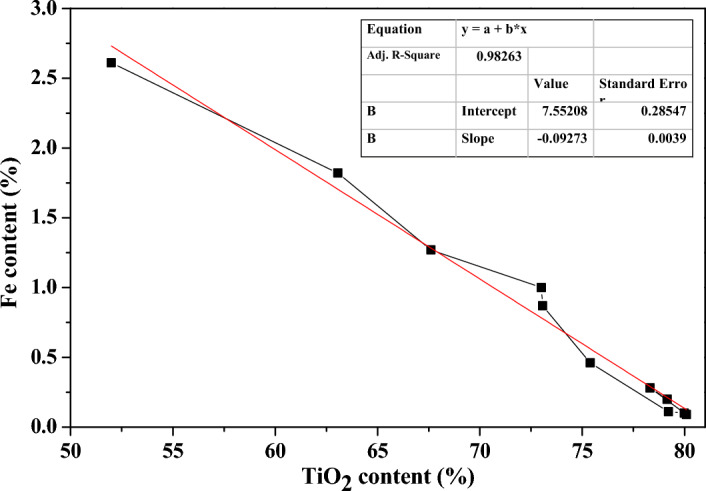
Relationships between ferrous ion content and TiO_2_ content.

The ferrous ion content (Fe %) in *MA* samples decreased significantly with the hydrolysis time increasing, as listed in Table 1, and the ferrous ion content basically remained at about 0.10% at hydrolysis time of 167–227 min, mainly due to the newly precipitated fine *MA* particles in this period were few, the adsorbed ferrous ion content changed little, and reached the smallest value of 0.09% at 227 min. After adding dilution water at 227 min, the amount of impurities adsorbed increased, and the ferrous ion content increased, due to the formation of some smaller *MA* particles with strong adsorption capacity. There was a nearly linear relationship between the ferrous ion content and the TiO_2_ content of the dried *MA* samples, as shown in Fig. 9, and the correlation coefficient *R*^*2*^ was of 0.98263, indicating that it had a good linear relationship, and the water content in *MA* was the key factor causing the change of ferrous ion content, because most ferrous ions were dissolved in the water adsorbed by *MA*.

The *MA* colloidal particles absorbed a large amount of water and impurities due to their bigger *S*_*BET*_, while impurities such as Fe^2+^ ion would be dissolved in the adsorbed water except for a small amount of them were adsorbed and bounded to *MA*. It could be seen that, with the extension of hydrolysis time, the grain size increased, the TiO_2_ content of *MA* gradually increased, the structure of *MA* became more compact, and its colloidal properties weakened. On the one hand, the adsorption ability of ferrous ion for *MA* on its surface was weakened, resulting in a decrease in the amount of ferrous ion adsorbed on its surface. On the other hand, the amount of water absorbed by *MA* was also reduced, resulting in a decrease in the number of dissolved ferrous ion in the adsorbed water. The two effects of structural changes in *MA* resulted in a decrease in its *Fe* content. The research results were consistent with the structural changes of *MA* reported in literature^27^. In order to obtain high quality *MA*, ultrasonic washing could be used and the pH value of the washing water could be adjusted to further save water and remove impurities such as ferrous ions^28^. In practice, to improve the purity of *MA* and the quality of titanium dioxide pigments, a feasible method is to properly increase the crystallinity of *MA* and the solid TiO_2_ content of filter cake, weaken the colloidal properties, and use higher pressure filtration device to reduce the moisture content of metatitanic acid and impurity content. The reduction of impurity content in *MA* would greatly save the water consumption in the subsequent washing and impurity removal process, and the lower moisture content of *MA* would also greatly save the energy consumption in the calcination process. And this would provide new ideas and ways to improve the clean production level of titanium dioxide production.

## Conclusions

Metatitanic acid was prepared from industrial TiOSO_4_ solution by extra-adding seeded thermal hydrolysis method. The hydrolysis process after gray point could be divided into the rapid hydrolysis stage and the slow hydrolysis maturation stage, and the hydrolysis degree changes conformed to Boltzmann model with good fitting. The structural evolution of the precipitated *MA* changed obviously as the hydrolysis proceeding, the content of TiO_2_ for *MA* gradually increased due to its more compact structure and weaker colloidal property, which was determined by the aggregation and particle adjustment of *MA* particles. The crystal size of *MA* increased significantly at the lower TiOSO_4_ concentration, and its lattice strain gradually decreased with the crystal growth and structure adjustment, which led that the average particle size was constantly adjusted and reduced. The aggregating and stacking of the precipitated *MA* particles formed the microporous and mesoporous structures, with the BET surface area larger than 265 m^2^/g. The Ti–O bond of metatitanic acid was mainly combined with sulfate and hydroxyl, and the pores were filled with sulfate and water. Ferrous ions mainly existed in *MA* by dissolving in the adsorbed water, a small amount existed in the form of adsorption and bonding. And the ferrous ion content decreased linearly with the increase of TiO_2_ content.

## Data Availability

All data generated or analyzed during this study are included in this manuscript.
